# The effect of eight yoga sessions on interoceptive accuracy, confidence and awareness in a sample of patients with eating disorder: A preliminary study

**DOI:** 10.1192/j.eurpsy.2021.946

**Published:** 2021-08-13

**Authors:** V. Nisticò, G. Boido, S. Bertelli, S. Anselmetti, M. Ischia, A. Priori, O. Gambini, B. Demartini

**Affiliations:** 1 “aldo Ravelli” Research Center For Neurotechnology And Experimental Brain Therapeutics, Università degli Studi di Milano, Milano, Italy; 2 Dipartimento Di Scienze Della Salute, Università degli Studi di Milano, Milano, Italy; 3 Unità Di Psichiatria Ii, ASST Santi Paolo e Carlo, Presidio San Paolo, Milano, Italy; 4 Nutrimente Onlus, Nutrimente Onlus, Milan, Italy; 5 Iii Clinica Neurologica, ASST Santi Paolo e Carlo, Presidio San Paolo, Milano, Italy

**Keywords:** eating disorders, yoga, Interoception, Heartbeat Counting Task

## Abstract

**Introduction:**

Previous research from our group showed that, after a single yoga class, Interoceptive Accuracy (IAc), tested through the Heartbeat Counting Task, improved in a group of Healthy Controls (HC), but not in a group of patients with Anorexia Nervosa (AN).

**Objectives:**

To evaluate three levels of interoception (accuracy, confidence (IC) and awareness (IAw)) before and after eight sessions of Yoga in a sample of patients with Eating Disorders (ED: AN, Bulimia Nervosa (BN) and Binge Eating Disorder (BED)).

**Methods:**

15 patients with ED were included. Before the first yoga session (T0) and 72 hours after the last session (T1), participants underwent: (i) the Heartbeat Counting Task for the evaluation of IAc, IC and IAw; (ii) a psychometric assessment evaluating depression, anxiety, body awareness, alexithymia, self-objectification and eating disorders symptomatology.

**Results:**

At T1, ED patients’ IAc appeared higher than at T0, but not IC and IAw. A trend towards significance (p = 0.055) emerged for the interaction effect between IAc and diagnosis, with BED patients having a higher increase of IAc at T1 than AN and BN patients. Significant correlations between IAc and Alexithymia, Anxiety and Depression emerged at T0, but were not maintained at T1.

**Conclusions:**

After a program of eight Yoga sessions, IAc in ED patients (but not IC and IAw) increases, especially in BED patients. Moreover, the improvement of IAc following the yoga course seems to be unrelated to the course of depressive, anxious and alexithymic symptoms of ED patients.

